# Influence of Residual Stress Field on the Fatigue Crack Propagation in Prestressing Steel Wires

**DOI:** 10.3390/ma8115400

**Published:** 2015-11-11

**Authors:** Jesús Toribio, Juan-Carlos Matos, Beatriz González, José Escuadra

**Affiliations:** Fracture and Structural Integrity Research Group, University of Salamanca, E.P.S., Campus Viriato, Avda. Requejo 33, 49022 Zamora, Spain; jcmatos@usal.es (J.-C.M.); bgonzalez@usal.es (B.G.); jeb@usal.es (J.E.)

**Keywords:** prestressing steel wires, semi-elliptical surface cracks, numerical modeling, Walker law, residual stress profile, fatigue crack propagation, crack front aspect ratio, preferential crack path

## Abstract

This paper deals with the effect of several residual stress profiles on the fatigue crack propagation in prestressing steel wires subjected to tension loading or bending moment. To this end, a computer program was developed to evaluate the crack front evolution on the basis of the Walker law. Results demonstrate that the absence of residual stresses makes the crack propagate towards a *preferential crack path*. When surface residual stresses are tensile and, correspondingly, core residual stresses are compressive, the fatigue crack fronts rapidly converge towards a *quasi-straight* *shape*. When surface residual stresses are compressive, with their corresponding tensile stresses in the core area, a *preferential crack path* also appears.

## 1. Introduction

Prestressing steel wires are manufactured by cold drawing a hot rolled pearlitic steel bar and have excellent mechanical properties making them very adequate components of prestressed concrete. The cold drawing process, apart from improving the mechanical properties (increase of yield stress, ultimate tensile strength, fracture toughness… [1]), induces strong tensile residual stresses at the wire surface and compressive ones in the core area of the wire due to non-uniform plastic strains [2].

Such residual stresses in prestressing steel wires were obtained using experimental methods [3,4] (neutron and X-ray diffraction techniques) and numerical procedures such as finite element methods (FEM) using the isotropic von Mises yield criterion [4,5] or a texture-based anisotropic yield locus [3]. In addition, the two micro-constituents of pearlite, ferrite and cementite, exhibit different residual stress profiles [6].

Changes in the die geometry for cold drawing (varying die angle) can modify the residual stress profile after cold drawing [5]. Frequently, prestressing steel wires are subjected to thermo-mechanical treatments (stretching and heating the material during a short time period) to eliminate or at least reduce after-drawing residual stresses [7]. These can also be diminished by using other thermo-mechanical processes, such as surface rolling, aiming to introduce compressive residual stresses in the surface area (and consequently tensile ones in the central region) [8].

Residual stresses generated after drawing in prestressing steels affect the measurement of important mechanical properties, e.g., those obtained performing a standard tension test [9], and also affect the phenomenon of stress relaxation in such materials [10]. In prestressing steel wires subjected to tensile cyclic loading, after-drawing residual stresses provoke the so-called *gull effect* over the crack front [11], which consists of retardation of fatigue crack growth in the areas of compressive residual stresses and acceleration in the tensile areas, thereby creating a gull shape in the crack front. Under cyclic loading it is understood that tensile residual stresses create only a slight increase in the crack propagation rate and compressive residual stresses create a big decrease in the crack propagation rate [12].

In the matter of hydrogen embrittlement of prestressing steel wires, residual stresses (especially the hydrostatic stress component) and plastic strains also affect the durability of the wires [13]. Surface treatments inducing compressive residual stresses in the near surface area clearly extend the life of the wires by delaying the diffusion of hydrogen towards the inner areas [14]. With regard to this topic, innovative design of the drawing die is proposed in [5] for reducing the residual stress and strain state in the cold drawn wires and, consequently, for improving the resistance to hydrogen embrittlement of prestressing steel wires.

Previous research [1,2,3,4,5,6,7,8,9,10,11,12,13,14,15] shows the relevant role of residual stress fields in the mechanics of materials (cyclic fatigue loading in particular, *cf.* [12,15]). Therefore, any attempt to analyze the effect of residual stresses on fatigue propagation in the wires should be welcome, on the basis of scientific reasons (to provide insight into the phenomenon) and from the technical viewpoint (to improve the behavior of the wires in real structures). In this framework, this paper describes a numerical procedure to analyze the influence of the residual stresses on the crack paths in prestressing steel wires subjected to fatigue cyclic loading. The studied variables are the residual stress profile, the level of residual stresses (compared to the applied stress), and the type of loading (tension or bending).

## 2. Numerical Procedure

In order to study how a crack propagates on the cross-section of a round bar under tension or bending cyclic loading, a computer program in the Java programming language was developed by the authors to determine the evolution of the crack front in a residual stress field.

### 2.1. Material

The analyzed material is cold drawn prestressing steel in wire form. It comes from a hot rolled bar with eutectoid composition and pearlitic microstructure that was drawn in seven steps until reaching a cumulative plastic strain ε^P^ = 1.57. Later the material was subjected to a thermo-mechanical treatment to release the residual stresses produced by the cold drawing procedure.

The mechanical properties of the steel are: Young’s modulus *E* = 209 GPa, yield strength σ_Y_ = 1480 MPa, and ultimate tensile strength σ_R_ = 1820 MPa. Its fatigue behavior was characterized by means of the Paris curve (with *R*-ratio = 0): exponent *m* = 3 and *C* = 4.1·10^−12^ (in the corresponding units for Δ*K* in MPam^1/2^ and d*a*/d*N* in m/cycle) [16].

### 2.2. Residual Stresses

Several profiles of residual stress (σ_res_) in the axial direction were used in the computations (this is the component of residual stresses trying to open the crack), satisfying the global equilibrium conditions over the transverse section of a wire:

(1) $$\int \int_{A}\sigma_{\text{res}}\text{d}A=\int_{0}^{2\text{π}}\int_{0}^{D/2}\sigma_{\text{res}}r\text{d}rd\text{θ}=0$$

where *A* is the transversal area, *r* the radial coordinate, θ the angular coordinate, and *D* the wire diameter.

**Figure 1:**
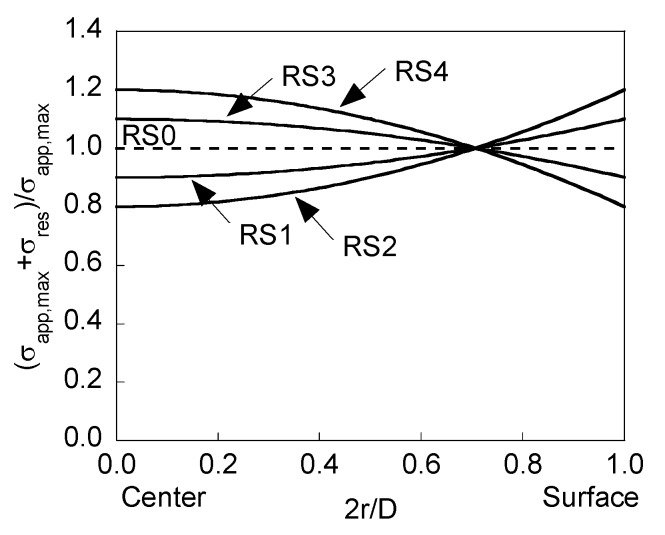
Stress profiles in cold drawn pearlitic steels.

Figure 1 shows the dimensionless stress profile (σ_app,max_ + σ_res_)/σ_app,max_ (maximum applied remote stress σ_app,max_ plus residual stress prior to cracking σ_res_ compared to the former) as a function of the dimensionless ratio 2*r*/*D*.

In this paper the performance of the prestressing steel wire free of residual stresses (RS0) is compared with that of wires with four theoretical (hypothetical) residual stress profiles of different intensity and sign (tensile or compressive nature). The four of them were derived from the same quadratic polynomial equation, *i.e.*, they have the same basic shape.

Profiles RS1 and RS2 (tensile stresses in the near-surface area, RS2 having twice the intensity of RS1) approximately correspond to those measured in prestressing steel wire after a thermo-mechanical treatment to reduce residual stresses after cold drawing [7].

On the other hand, profiles RS3 and RS4 (compressive stresses in the near-surface area, RS4 having twice the intensity of RS3) represent the opposite situation (symmetrical profile) to that previously analyzed in curves RS1 and RS2.

### 2.3. Numerical Modeling

**Figure 2:**
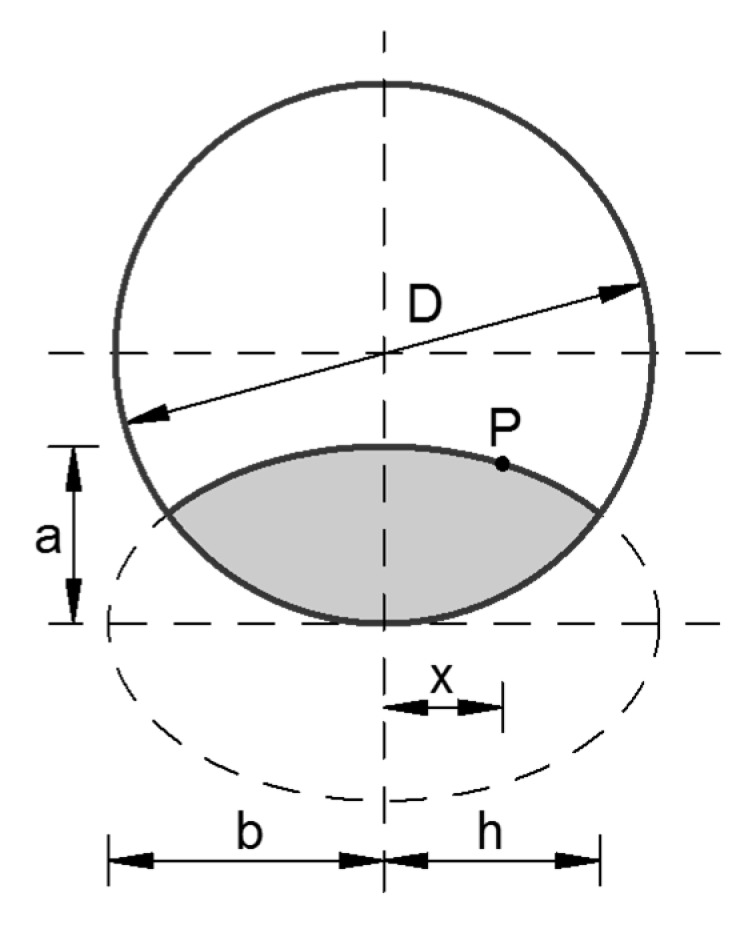
Elliptical crack model.

Surface cracks contained in the transverse section of the wire were characterized as part of an ellipse with its center in the wire surface of diameter *D* (Figure 2). Parameters defining the geometry of the ellipse are the crack depth *a* (associated with one semiaxis of the ellipse) and the other semiaxis of the ellipse *b*. Each point *P* at the crack front is determined by the coordinate *x*, *h* being the coordinate linked with the point located at the wire surface.

Fatigue crack advance under cyclic tension loading or cyclic bending moment is analyzed by using the Paris law [17]:

(2) $$\frac{da}{dN}=C\Delta K^{\text{m}}$$

modified by means of the Walker equation (Paris model by incorporating the effect of the *R*-ratio, *i.e.*, it takes into account the influence of mean stress) [18]:

(3) $$\frac{da}{dN}=C{\left(\frac{\Delta K}{{\left(1-R\right)}^{\left(1-\gamma \right)}}\right)}^{\text{m}}$$

where *C* and *m* are the Paris parameters for the *R*-ratio = 0 and γ is the Walker exponent. This parameter was supposed to be zero, on assuming that the *R*-ratio,

(4) $$R=\frac{\sigma_{\text{app,min}}+\sigma_{\text{res}}}{\sigma_{\text{app,max}}+\sigma_{\text{res}}}$$

is always lower than zero (the compressive portion of the applied stress is ignored by most of the commonly used models of fatigue crack propagation), so that the dependence on the *R*-ratio of the crack growth rate is very pronounced and the maximum stress intensity factor (SIF) *K*_max_ is the crack driving force [19,20]:

(5) $$\frac{da}{dN}=C{\left(K_{\text{max}}\right)}^{\text{m}}$$

The maximum SIF *K*_max_ is given by:

(6) $$K_{\max}=Y\sigma_{\max}\sqrt{\text{π}a}$$

*Y* being the dimensionless SIF. With regard to σ_max_, it is obtained using the superposition principle as:

(7) $$\sigma_{\text{max}}=\sigma_{\text{app,max}}+\sigma_{\text{res}}$$

The maximum applied remote stress on the round bar σ_app,max_ for tension loading is calculated as:

(8) $$\sigma_{\text{app,max}}=\frac{4F}{\text{π}D^{2}}$$

*F* being the maximum axial force and *D* the diameter. The stress σ_app,max_ for bending is given by:

(9) $$\sigma_{\text{app,max}}=\frac{32M}{\text{π}D^{3}}$$

where *M* is the maximum applied moment.

The *K*-solution used in this paper is that proposed by Shin and Cai [21] with three parameters, which are the relative crack depth *a*/*D*, the crack aspect ratio *a*/*b*, and the position at the crack front *x*/*h* (Figure 2). The dimensionless SIF *Y* is defined as a function of the coefficient *M*_ijk_ for tension with free sample ends [21]:

(10) $$Y=\sum_{i=0}^{2}\sum_{j=0}^{7}\sum_{k=0}^{2}M_{\text{ijk}}{\left(\frac{a}{b}\right)}^{\text{i}}{\left(\frac{a}{D}\right)}^{\text{j}}{\left(\frac{x}{h}\right)}^{\text{k}}$$

and as a function of the coefficient *N*_ijk_ for bending [21]:

(11) $$Y=\sum_{i=0}^{2}\sum_{j=0}^{6}\sum_{k=0}^{2}N_{\text{ijk}}{\left(\frac{a}{b}\right)}^{\text{i}}{\left(\frac{a}{D}\right)}^{\text{j}}{\left(\frac{x}{h}\right)}^{\text{k}}$$

The crack front (characterized as an ellipse [22]) was discretized as a set of points (obtained by dividing the ellipse in *z* parts of equal longitude by applying the Simpson rule). The advance at each point of the crack (*i*) is perpendicular to the crack front. The point at the crack front associated with the maximum value of the product *Y*σ_max_, (*Y*σ_max_){max}, is advanced as a fixed value ∆*a*{max} and the rest of the points over the crack front consider the Walker law as follows:

(12) $$\Delta a\left(i\right)=\Delta a\left\{\text{max}\right\}{\left[\frac{Y\left(i\right)\sigma_{\text{max}}\left(i\right)}{\left(Y\sigma_{\text{max}}\right)\left\{\text{max}\right\}}\right]}^{\text{m}}$$

The newly obtained points were fitted to a new ellipse and the process is repeated until the desired crack length is reached.

On the basis of the proposed model, there are two key aspects in fatigue crack propagation: changes in dimensionless SIF and maximum stress (due to residual stresses) over the crack front. The first is determined by the type of loading and both depend on the crack geometry, so both have an influence on each other because the two are dependent on the crack shape while at the same time they determine changes in the crack geometry itself.

## 3. Numerical Results and Discussion

**Figure 3:**
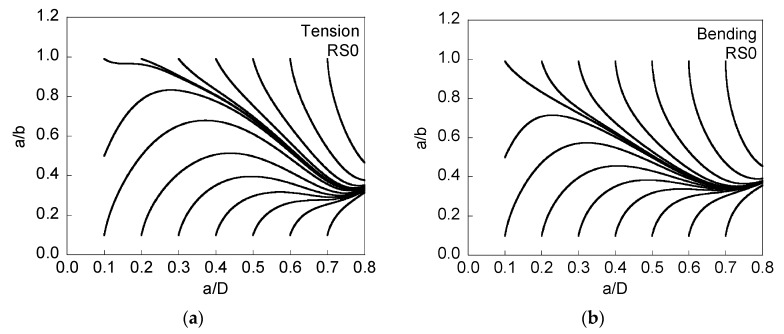
Evolution of the aspect ratio *a*/*b* with crack growth (represented by the relative crack depth *a*/*D*) for RS0, cyclic tension loading (**a**); and cyclic bending moment (**b**).

**Figure 4:**
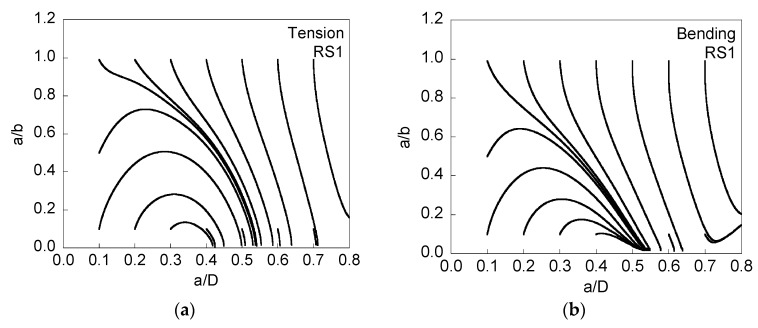
Evolution of the aspect ratio *a*/*b* with crack growth (represented by the relative crack depth *a*/*D*) for RS1, cyclic tension loading (**a**); and cyclic bending moment (**b**).

**Figure 5:**
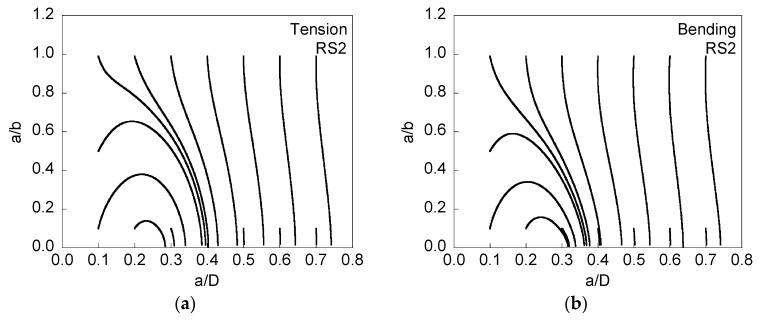
Evolution of the aspect ratio *a*/*b* with crack growth (represented by the relative crack depth *a*/*D*) for RS2, cyclic tension loading (**a**); and cyclic bending moment (**b**).

**Figure 6:**
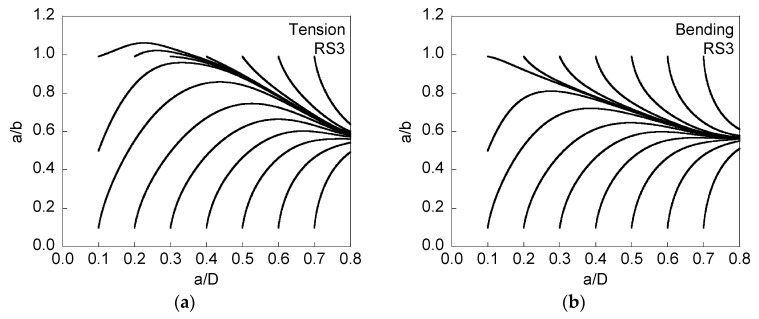
Evolution of the aspect ratio *a*/*b* with crack growth (represented by the relative crack depth *a*/*D*) for RS3, cyclic tension loading (**a**); and cyclic bending moment (**b**).

**Figure 7:**
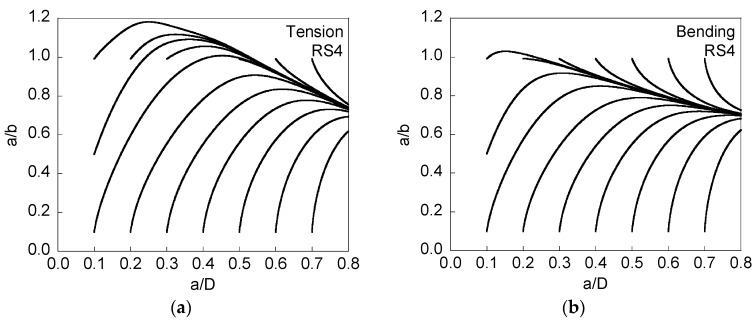
Evolution of the aspect ratio *a*/*b* with crack growth (represented by the relative crack depth *a*/*D*) for RS4, cyclic tension loading (**a**); and cyclic bending moment (**b**).

A convergence study was performed to determine the number of points *z* in which the crack front is discretized and the maximum crack growth increment ∆*a*{max}. Figure 3, Figure 4, Figure 5, Figure 6 and Figure 7 show the evolution of the crack front during fatigue crack propagation from different initial geometries (semi-circular, initial *a*/*b* = 1; quasi-straight, initial *a*/*b* = 0.1).

Figure 3 corresponds to the case in which residual stresses are null, the plots being independent of the maximum applied stress σ_max_, because, in the absence of any residual stress (σ_res_ = 0), only the externally maximum applied stress σ_app,max_ (common for all points of the crack front) controls the crack advance. This figure shows how, starting from different initial geometries, all plots converge towards a *preferential cracking path* in which the aspect ratio *a*/*b* is higher under tension than under bending (in both loading cases for a given relative crack depth *a*/*D*), with the exception of very deep cracks (*a*/*D* > 0.7). The reason why the aspect ratio of the propagating cracks is different under tension loading than under bending moment is due to the distinct dimensionless SIF values at different points of the crack front for tension and bending [21].

With regard to the preferential cracking path, higher aspect ratio values also appear in two cases: (i) when the wire is subjected to tensile loading with constrained ends (if compared with the case of free ends analyzed in the present paper) [23]; (ii) when the Paris exponent *m* decreases [23,24]. The preferential crack path approaches that starting from a very shallow quasi-circular initial crack.

Figure 4 and Figure 5 present the evolution of the crack front when tensile residual stresses are presented at the wire surface (and the corresponding compressive stresses in the core), and Figure 5 is associated with twice the level of the residual stresses of Figure 4.

When residual stresses are tensile at the surface and compressive in the core area, the growing cracks tend rapidly towards a *quasi-straight crack front* *a*/*b* ~ 0, the instant at which the modeling is stopped because the elliptical geometry of the crack front disappears. The approach of the different curves to this straight front takes place for lower relative crack advancement as the intensity of the stress distribution (see Figure 1) increases, *i.e.*, it is quicker for RS2 than for RS1. In addition, such a stress profile seems to have a bigger influence on crack propagation for tension loading than for bending moment, e.g., Figure 4b that shows how three curves starting from very deep initial cracks do not reach the straight geometry during propagation, whereas in Figure 4a this only happens for a single curve.

Figure 6 and Figure 7 present the evolution of the crack front when compressive residual stresses are presented at the wire surface (and the corresponding tensile stresses in the core), and Figure 7 is associated with twice the level of the residual stresses of Figure 6.

When residual stresses are compressive at the surface and tensile in the core area, the growing cracks become closer (from different initial geometries) during *crack growth towards a* *preferential cracking path* (as in the absence of residual stresses), with such a path exhibiting a greater aspect ratio *a*/*b* as the intensity of the stress distribution (see Figure 1) increases, *i.e.*, it is higher for RS4 than for RS3. This rise of the preferential cracking path for RS3 and RS4 with respect to RS0 is higher as the crack depth increases and, for a given residual stress profile, the crack aspect ratio *a*/*b* is higher in tension than in bending.

Compressive residual stresses induce fatigue crack growth retardation, while tensile residual stresses accelerate cracking. When the compressive ones are located near the surface and the tensile ones in the central area, the evident consequence is that the crack advances towards higher aspect ratios. When the situation is the opposite (tensions near the surface and compressions at the center) then the aspect ratio decreases, very quickly, during crack advance.

## 4. Conclusions

The following conclusions can be drawn on the basis of the numerical analysis performed in the present work:

(i) The absence of residual stresses makes the crack propagate towards a *preferential crack path*. In a plot *a*/*b*−*a*/*D*, such a path is higher (greater *a*/*b* for a given *a*/*D*) in tension than in bending.
(ii) A residual stress profile with tensions in the vicinity of the wire surface and compressions in the central area makes the crack propagate towards a *quasi-straight crack front*. Such a profile seems to affect crack propagation more markedly in tension than in bending.
(iii) A residual stress profile with compressions in the vicinity of the wire surface and tensions in the central area makes the crack propagate towards a *preferential crack path* (as in the case of material free of residual stresses). In a plot *a*/*b*−*a*/*D*, such a path is higher (greater *a*/*b* for a given *a*/*D*) in tension than in bending, and higher than in the absence of any residual stress.
(iv) With regard to the effect of the intensity of residual stresses present in the material, the stress level enhances the aforementioned phenomena of approaching a quasi-straight crack front and raising the level of the preferential crack path, *i.e.*, curve RS2 produces a quicker straightening than curve RS1 and curve RS4 raises the level of the preferential crack path more than curve RS3 (and both raise such a level when they are compared with curve RS0 without residual stresses).
